# Supplementary material for: Guanidinocalix[5]arene for sensitive fluorescence detection and magnetic removal of perfluorinated pollutants
Source: Nat Commun. 2019 Dec 17;10:5762. doi: 10.1038/s41467-019-13775-1 (PMC6917741; doi:10.1038/s41467-019-13775-1)
Supplement: Supplementary file 1 — Supplementary Information [file 41467_2019_13775_MOESM1_ESM.pdf]

Supplementary Information

for

Guanidinocalix[5]arene for Sensitive Fluorescence Detection and Magnetic Removal of Perfluorinated Pollutants

Zhe Zheng,[1] Huijuan Yu,[2] Wen-Chao Geng,[1] Xin-Yue Hu,[1] Yu-Ying Wang,[1] Zhihao Li,[1] Yuefei Wang[2] and Dong-Sheng Guo[1]

[1] College of Chemistry, State Key Laboratory of Elemento-Organic Chemistry, Key Laboratory of Functional Polymer Materials (Ministry of Education), Nankai University, Tianjin 300071, China.

[2] Institute of Traditional Chinese Medicine, Tianjin University of Traditional Chinese Medicine, Tianjin 301617, China.

Corresponding authors: dshguo@nankai.edu.cn (Dong-Sheng Guo)

1. Supplementary Notes

Supplementary Note 1 | Theoretical calculations. All density functional theory (DFT) calculations were carried out using the Gaussian 16 program.[1] SMD solvation model[2] was used in all calculations. The geometry optimizations were carried out using B3LYP/6-31G(d)[3] level of theory with Grimme's D3 dispersion correction.[4] Default convergence criteria were used for the optimization with Gaussian 16. Independent gradient model (IGM) analysis was derived by using the Multiwfn software.[5] Molecular graphs were visualized by the visual molecular dynamic program.[6]

Supplementary Note 2 | Water samples with solid-phase extraction method. Water samples from the tap, Mati Lake and Haihe River were filtered through a nylon film (0.45 μm) before experiments. The aliquots of blank tap water samples and those spiked with 50 ng L^{-1} of PFOS or PFOA were extracted with HLB cartridges. (6 cc, 150 mg; Waters Corp. Milford, U.S.A.). First, the cartridges were activated and conditioned with 5 mL methanol and 5 mL water. Second, the water samples (250 mL) were passed through the wet cartridges. Third, the columns rinsed with 5 mL sodium acetate buffer (25 mM) and 10 mL methanol, and the cartridges dried for 30 min by N_2. Then, elution was performed with 2% ammonium hydroxide in methanol (7 mL). Finally, the eluate was then concentrated to 250 μL for fluorescence displacement assay.

Supplementary Note 3 | Visual determination of PFOS and PFOA using a smartphone and a handheld UV lamp. GC5A-6C•Fl (8.0/10.0 μM) reporter pair in HEPES buffer was mixed up with various concentrations of PFOS and PFOA for detection. The solution (2.0 mL) of each group was added into a polypropylene centrifuge tube (BBI Life Sciences, 2.5 mL), followed by exciting with a 254 nm UV lamp. Then, colour change could be taken by an iPhone 7 and the images were handled with a colour scanning application from Apple Store (World of Color, Maarten Zonneveld). For other models of smartphones, the present calibration curves for iPhone 7 may not work exactly and new calibration curves may need be set up. RGB intensities were displayed on the screen and G value was extracted for determination of PFOS and

PFOA.

Supplementary Note 4 | Preparation of the pegylated GC5A-12C nanoparticle. GC5A-12C (100 μM) and PEG-12C (50 μM) were dissolved in mixture solution of methanol and chloroform. After removal of solvent under reduced pressure for 12 h, the residue was hydrated in HEPES buffer (10 mM, pH = 7.4) by sonication at 80 °C for 3-5 h.

Supplementary Note 5 | Preparation of the hybrid calixarene nanoparticle (MNP@GC5A-12C). A mixture of MNP (0.2 mg mL^{-1}), GC5A-12C (100 μM) and PEG-12C (10 μM) were dissolved in methanol and chloroform. After removal of solvent under reduced pressure for 12 h, the residue was hydrated in water by sonication at 80 °C for 3-5 h.

Supplementary Note 6 | Quantification of PFOS and PFOA from the absorption studies. Quantification of PFOS (50 ng mL^{-1} to 4000 ng mL^{-1}) and PFOA (100 ng mL^{-1} to 1000 ng mL^{-1}) from the absorption experiments at $[PFOS]_0 = [PFOA]_0 = 1000$ ng mL^{-1} were performed by means of UPLC-ESI-MS/MS.

Equipped with binary solvent manager, sample manager and column oven, ACQUITYTM UPLC I-Class system (Waters, Milford, MA, USA) was employed to perform chromatographic analysis, which was controlled by MassLynx V4.1 software (Waters, Milford, MA, USA). Carried on ACQUITYTM UPLC® BEH C18 column (2.1 mm × 100 mm, 1.7 μm) at 50 °C, the chromatographic separation was accomplished by mobile phase consisting of 2 mM ammonium acetate aqueous solution (v/v) (A) and 95% methanol (B) in an isocratic elution. The flow rate of mobile phase was set at 0.3 mL min^{-1} and the injection volume was 2 μL.

The UPLC system was coupled to Waters Xevo TQ-S triple quadrupole mass spectrometer (Waters, Milford, MA, USA) equipped with an electrospray ionization source operating in the negative ion mode. The optimized parameters were as follows: capillary voltage at − 2.0 kV, source temperature at 150 °C, desolvation temperature at 500 °C, desolvation gas flow at 1000 L h^{-1}, cone gas flow at 150 L h^{-1}, and nebulizer

gas flow at 7.0 bar. The multiple reaction monitoring (MRM) acquisition mode was performed to detect the focused compounds after optimizing the parameter of each compound, such as cone voltage and collision energy. The cone voltage and collision energy of the detected compounds were listed in Supplementary Table 1. All the data were acquired and processed by MassLynx V4.1 software (Waters, Milford, MA, USA).

Matrix-matched calibration standards were prepared with different concentrations for external calibration. Analytes were quantified from calibration standards based on the PFOS and PFOA responses by linear least-squares regression. The detailed results were summarized in Supplementary Table 2. Calibration curves were run at the beginning of the analytical run. Instrument blanks were run before and after the calibration curve and each batch of triplicate samples.

2. Supplementary Tables

Supplementary Table 1 | The detecting parameters of compounds in MRM mode for LC-MS/MS analysis.

Compound	Formula	t_R (min)	MRM Transitions	Cone Voltage (V)	Collision Energy (eV)
PFOS	$C_8F_{17}SO_3K$	1.73	498.7→80.0	94	36
PFOA	$C_8HF_{15}O_2$	1.68	368.8→168.9	48	14

Supplementary Table 2 | Analytical data required for PFOS and PFOA quantification.

**Compound**	**Regression Equations (*n*=3)**	***r***	**Linear Range (ng mL^{-1})**	**Limit of detection (ng mL^{-1})**
PFOS	$y = 2858.3\,x - 77820.0$	0.9960	50 – 4000	1.0
PFOA	$y = 2702.2\,x - 57930.1$	0.9950	100 – 1000	0.5

Supplementary Table 3 | Reported binding affinities (K_a) of other supramolecular hosts to PFOS.

Supramolecular Host	K_a	Ref
β-CD[a]	$(8.85 \pm 4.4) \times 10^4$ M^{-1}	7
RAMEB[b]	$(3.55 \pm 1.4) \times 10^4$ M^{-1}	7
DM-*β*-CD[c]	$(2.49 \pm 0.81) \times 10^4$ M^{-1}	7
TM-*β*-CD[d]	$(2.48 \pm 1.7) \times 10^4$ M^{-1}	7
HM-*β*-CD[e]	$(3.59 \pm 1.1) \times 10^4$ M^{-1}	7
Tripodal fluorous amide host[f]	1.4×10^3 M^{-1}	8
Amide groups and fluorous ponytails modified calix[4]arene[g]	$< 10^4$ M^{-1}	9

[a] *β*-CD: *β*-cyclodextrin. [b] RAMEB: 2,3,6-randomly methylated *β*-CD. [c] DM-*β*-CD: 2,6-di-*O*-methyl *β*-CD. [d] TM-*β*-CD: 2,3,6-tri-*O*-methyl *β*-CD. [e] HP: 6-*O*-2-hydroxypropyl *β*-CD. [f] Tripodal fluorous amide host: *N*,*N'*,*N''*-(2,4,6-triethylbenzene-1,3,5-triyl)tris(methylene)tris-(2,2,2-trifluoroacetamide). [g] Amide groups and fluorous ponytails modified calix[4]arene: [2,2',2'',2'''-((15,35,55,75-tetra-*tert*-butyl-1,3,5,7(1,3)-tetrabenzenacyclooctaphane-12,32,52,72-tetrayl)tetrakis(oxy))tetrakis(*N*-(3,3,4,4,5,5,6,6,7,7,8,8,8-tridecafluorooctyl)acetamide)].

Supplementary Table 4 | Determination of PFOS and PFOA in different water samples by indicator displacement assay.

Sample	PFOS		PFOA	
	Recovery (%)	s.d. (%)	Recovery (%)	s.d. (%)
Tap water (Beijing)	100.7	10.5	95.0	7.3
Tap water (Tianjin)	92.0	7.3	97.7	8.0
Tap water (Hebei)	90.3	0.5	95.7	3.3
Haihe River	101.0	12.8	102.7	9.4

3. Supplementary Figures

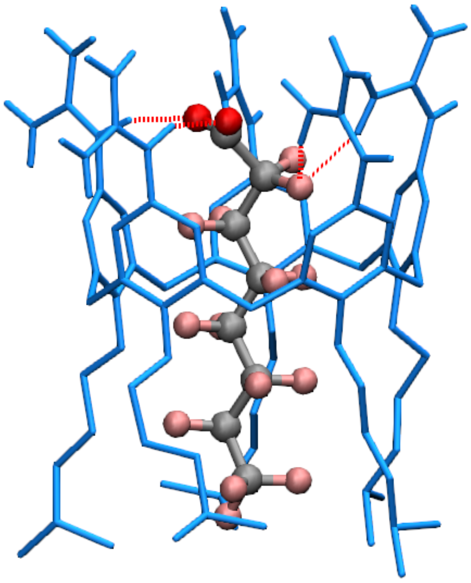

Supplementary Figure 1 | Optimized structure of the GC5A-6C•PFOA complex at the B3LYP-D3/6-31G(d)/SMD(water) level of theory. Evident hydrogen bonds are shown by red dashed lines.

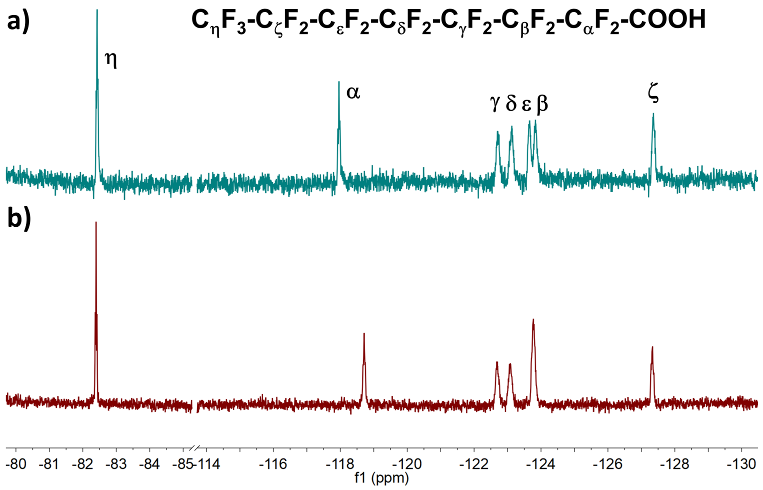

Supplementary Figure 2 | ^{19}F NMR (376 MHz) spectra of (a) PFOA (0.1 mM) and (b) PFOA (0.1 mM) with addition of GC5A-6C (1.0 mM) in CD_3OD at 25 °C. The ^{19}F NMR spectra were referenced externally to TFE (δ −78.84).

Calixarenes are non-emissive in these experiments, which indicates the fluorescence signals come from the host-guest complex but not the host (Supplementary Fig. 3).

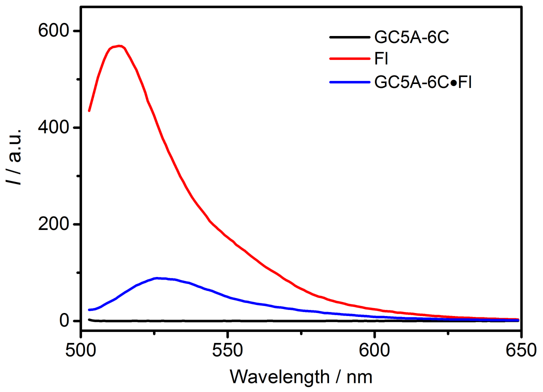

Supplementary Figure 3 | Fluorescence spectra of GC5A-6C, Fl and GC5A-6C•Fl complex. Fluorescence spectra of GC5A-6C (10.0 μM), Fl (1.0 μM) and GC5A-6C•Fl complex (10.0/1.0 μM) in HEPES buffer (10 mM, pH = 7.4) at 25 °C, λ_{ex} = 500 nm.

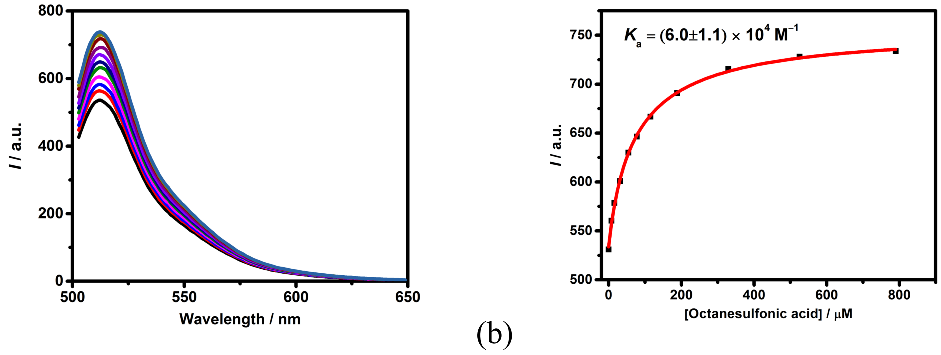

Supplementary Figure 4 | Competitive fluorescence titration and the associated titration curve of GC5A-6C•Fl reporter pair and octanesulfonic acid. (a) Competitive fluorescence titration of GC5A-6C•Fl (0.4/0.5 μM) with octanesulfonic acid (up to 791 μM), λ_{ex} = 500 nm. (b) The associated titration curve at λ_{em} = 513 nm and fit according to a 1:1 competitive binding model. All experiments were performed in HEPES buffer (10 mM, pH = 7.4) at 25 °C. Data represent mean ± s.d. (n = 3 independent experiments).

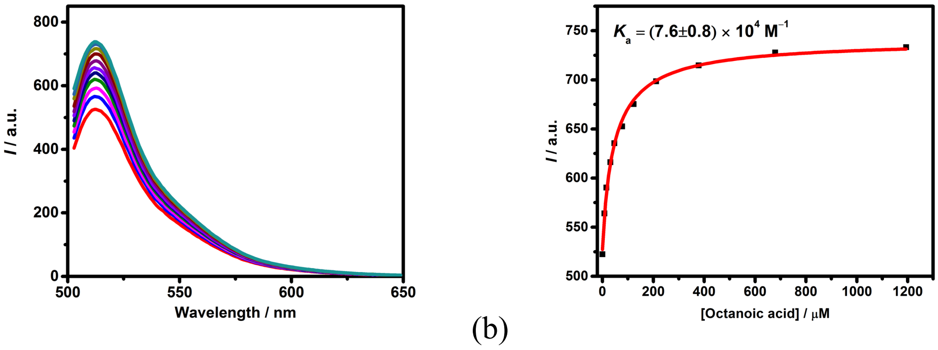

(a) (b)

Supplementary Figure 5 | Competitive fluorescence titration and the associated titration curve of GC5A-6C•Fl reporter pair and octanoic acid. (a) Competitive fluorescence titration of GC5A-6C•Fl (0.4/0.5 μM) with octanoic acid (up to 1190 μM), λ_{ex} = 500 nm. (b) The associated titration curve at λ_{em} = 513 nm and fit according to a 1:1 competitive binding model. All experiments were performed in HEPES buffer (10 mM, pH = 7.4) at 25 °C. Data represent mean ± s.d. (n = 3 independent experiments).

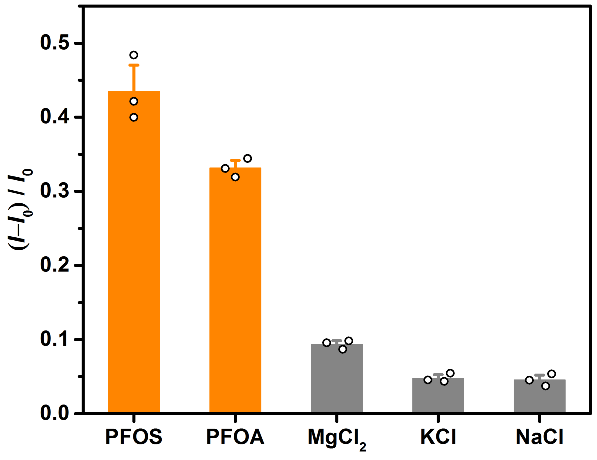

Supplementary Figure 6 | Influence of salt concentrations to selective detection of PFOS and PFOA. Fluorescence responses of GC5A-6C•Fl (0.8/1.0 μM) after adding PFOS, PFOA (0.8 μM) and interfering species (800 μM). I and I_0 are the intensities of fluorescence of the GC5A-6C•Fl reporter pair with and without the guest molecules, respectively. All experiments were performed in HEPES buffer (10 mM, pH = 7.4) at 25 °C, λ_{ex} = 500 nm. Error bars represent mean ± s.d. (n = 3 independent experiments).

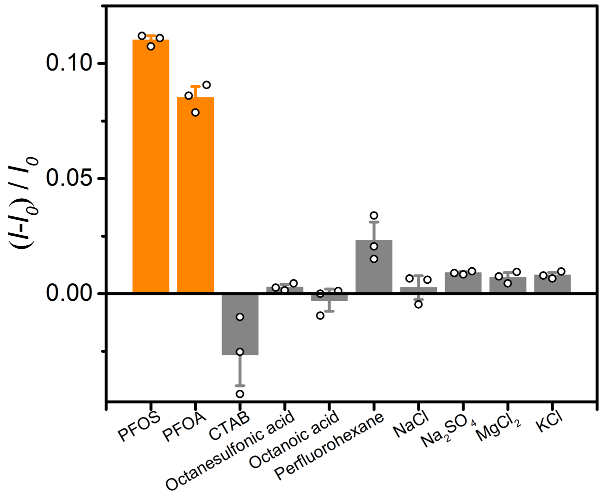

Supplementary Figure 7 | Selective detection of PFOS and PFOA in waste water. Fluorescence responses of GC5A-6C•Fl (10.0/1.0 μM) after adding PFOS, PFOA and interfering species (10.0 μM). *I* and I_0 are the intensities of fluorescence of the GC5A-6C•Fl reporter pair with and without the guest molecules, respectively. All experiments were performed in highly contaminated water at 25 °C, λ_{ex} = 500 nm, and λ_{em} = 513 nm. The CTAB is hexadecyltrimethylammonium bromide. Error bars represent mean ± s.d. (*n* = 3 independent experiments).

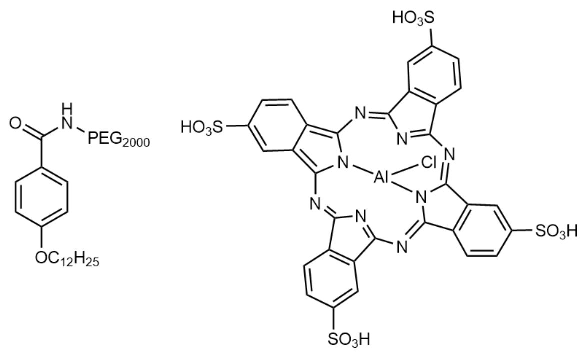

PEG-12C AlPcS$_4$

Supplementary Figure 8 | Structures of AlPcS$_4$ and PEG-12C.

Fl was also screened as reporter dye and the binding affinity with pegylated GC5A-12C nanoparticle was fitted as (5.3 ± 0.9) × 10^6 M^{-1}.

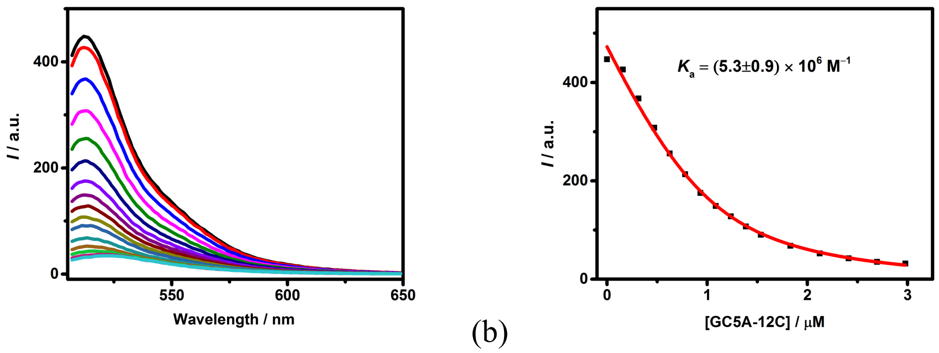

(a) (b)

Supplementary Figure 9 | Fluorescence titration and the associated titration curve of Fl and GC5A-12C. (a) Direct fluorescence titration of Fl (1.0 μM) with pegylated GC5A-12C nanoparticle (up to 2.7 μM), λ_{ex} = 500 nm. (b) The associated titration curve at λ_{em} = 513 nm and fit according to a 1:1 binding stoichiometry. All experiments were performed in HEPES buffer (10 mM, pH = 7.4) at 25 °C. Data represent mean ± s.d. (*n* = 3 independent experiments).

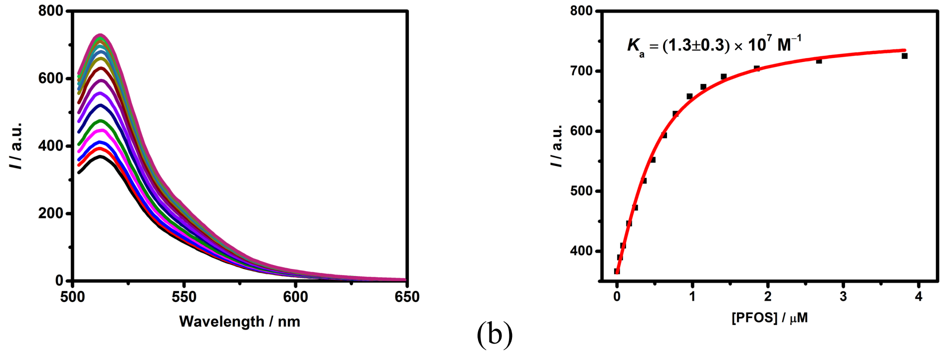

(a) (b)

Supplementary Figure 10 | Competitive fluorescence titration and the associated titration curve of GC5A-12C•Fl reporter pair and PFOS. (a) Competitive fluorescence titration of GC5A-12C•Fl (0.4/0.5 μM) with PFOS (up to 3.8 μM), λ_{ex} = 500 nm. (b) The associated titration curve at λ_{em} = 513 nm and fit according to a 1:1 competitive binding model. All experiments were performed in HEPES buffer (10 mM, pH = 7.4) at 25 °C. Data represent mean ± s.d. (*n* = 3 independent experiments).

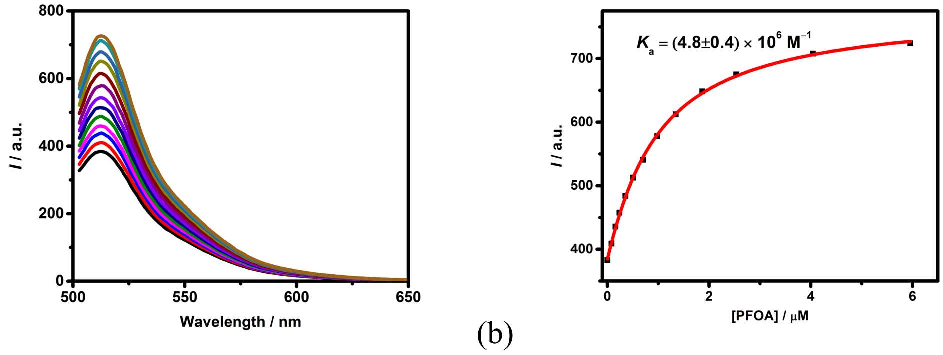

(a) (b)

Supplementary Figure 11 | Competitive fluorescence titration and the associated titration curve of GC5A-12C•Fl reporter pair and PFOA. (a) Competitive fluorescence titration of GC5A-12C•Fl (0.4/0.5 μM) with PFOA (up to 6.0 μM), λ_{ex} = 500 nm. (b) The associated titration curve at λ_{em} = 513 nm and fit according to a 1:1 competitive binding model. All experiments were performed in HEPES buffer (10 mM, pH = 7.4) at 25 °C. Data represent mean ± s.d. (n = 3 independent experiments).

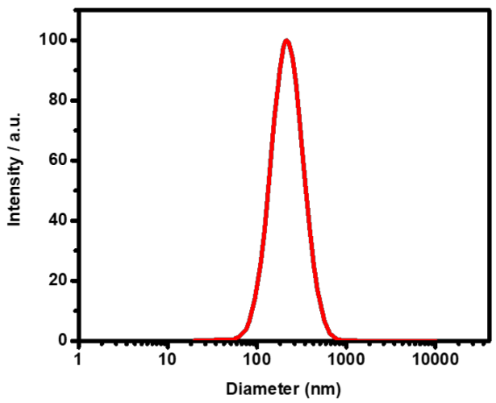

Supplementary Figure 12 | DLS data of MNP@GC5A-12C in water at 25 °C. ([GC5A-12C] = 100 μM; [PEG-12C] = 10 μM, [MNP] = 0.2 mg mL^{-1}).

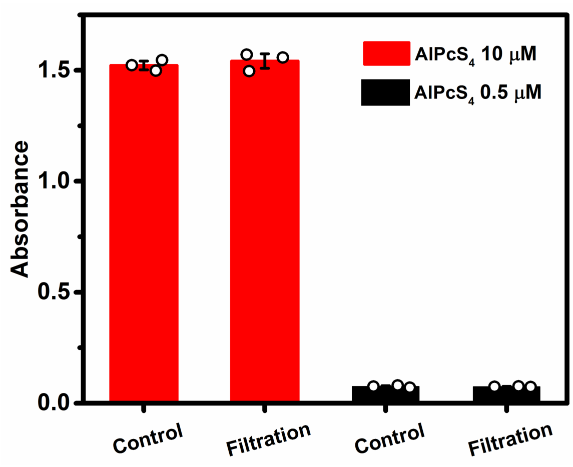

Supplementary Figure 13 | Two concentrations of $AlPcS_4$ (10 μM and 0.5 μM) with only filtration procedure detected by UV-Vis experiments. The $AlPcS_4$ without filtration procedure as control. Error bars represent mean ± s.d. ($n = 3$ independent experiments).

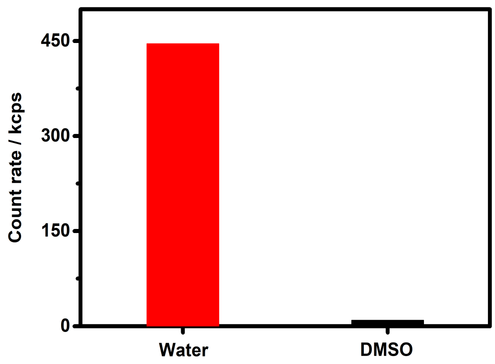

Supplementary Figure 14 | Scattering intensity of the pegylated GC5A-12C nanoparticle in water and DMSO at 25 °C. ([GC5A-12C] = 100 μM; [PEG-12C] = 50 μM).

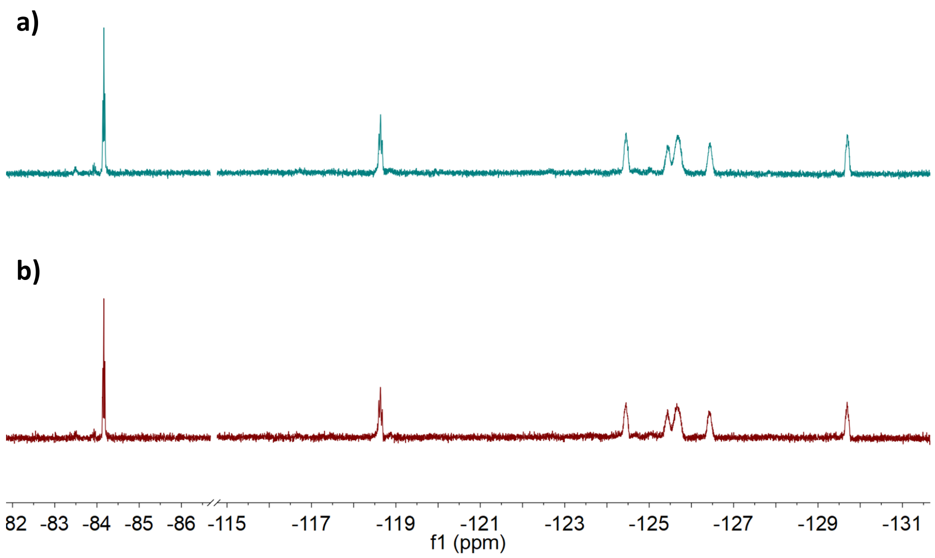

Supplementary Figure 15 | ^{19}F NMR (376 MHz) spectra of (a) PFOS (0.5 mM) with addition of GC5A-12C (0.5 mM) and (b) PFOS (0.5 mM) in DMSO-d_6 at 25 °C. The ^{19}F NMR spectra were referenced externally to TFE (δ −75.22).

4. Supplementary References

1. Gaussian 16, Revision A.03, M. J. Frisch, G. W. Trucks, H. B. Schlegel, G. E. Scuseria, M. A. Robb, J. R. Cheeseman, G. Scalmani, V. Barone, G. A. Petersson, H. Nakatsuji, X. Li, M. Caricato, A. V. Marenich, J. Bloino, B. G. Janesko, R. Gomperts, B. Mennucci, H. P. Hratchian, J. V. Ortiz, A. F. Izmaylov, J. L. Sonnenberg, D. Williams-Young, F. Ding, F. Lipparini, F. Egidi, J. Goings, B. Peng, A. Petrone, T. Henderson, D. Ranasinghe, V. G. Zakrzewski, J. Gao, N. Rega, G. Zheng, W. Liang, M. Hada, M. Ehara, K. Toyota, R. Fukuda, J. Hasegawa, M. Ishida, T. Nakajima, Y. Honda, O. Kitao, H. Nakai, T. Vreven, K. Throssell, J. A. Montgomery, Jr., J. E. Peralta, F. Ogliaro, M. J. Bearpark, J. J. Heyd, E. N. Brothers, K. N. Kudin, V. N. Staroverov, T. A. Keith, R. Kobayashi, J. Normand, K. Raghavachari, A. P. Rendell, J. C. Burant, S. S. Iyengar, J. Tomasi, M. Cossi, J. M. Millam, M. Klene, C. Adamo, R. Cammi, J. W. Ochterski, R. L. Martin, K. Morokuma, O. Farkas, J. B. Foresman, and D. J. Fox, Gaussian, Inc., Wallingford CT, 2016.
2. Marenich, A. V., Cramer, C. J. & Truhlar, D. G. Universal solvation model based on solute electron density and on a continuum model of the solvent defined by the bulk dielectric constant and atomic surface tensions. *J. Phys. Chem. B* **113,** 6378-6396 (2009).
3. Stephens, P. J., Devlin, F. J., Chabalowski, C. F. & Frisch, M. J. Ab initio calculation of vibrational absorption and circular dichroism spectra using density functional force fields. *J. Phys. Chem.* **98,** 11623-11627 (1994).
4. Grimme, S., Antony, J., Ehrlich, S. & Krieg, H. A consistent and accurate ab initio parametrization of density functional dispersion correction (DFT-D) for the 94 elements H-Pu. *J. Chem. Phys.* **132,** 154104 (2010).
5. Lu, T. & Chen, F. Multiwfn: a multifunctional wavefunction analyzer. *J. Comput. Chem.* **33,** 580-592 (2012).
6. Humphrey, W., Dalke, A. & Schulten, K. VMD: Visual molecular dynamics. *J. Mol. Graphics* **14,** 33-38 (1996).
7. Wilson, L. D. & Verrall, R. E. ^{19}F and ^{1}H NMR investigation of cyclodextrin/fluorocarbon alkyl carboxylate surfactant inclusion complexes. *Langmuir* **14,** 4710-4717 (1998).
8. Gavette, J. V., McGrath, J. M., Spuches, A. M., Sargent, A. L. & Allen, W. E. Fluorous effects in amide-based receptors for anions. *J. Org. Chem.* **74,** 3706-3710 (2009).
9. Omorodion, H., Palenzuela, M., Ruether, M., Twamley, B., Platts, J. A. & Baker, R. J. A rationally designed perfluorinated host for the extraction of PFOA from water utilising non-covalent interactions. *New J. Chem.* **42,** 7956-7968 (2018).
